# Identifying Over- and Underfunded Diseases by Comparing National Institutes of Health Funding for Skin Disease Research With US Skin Disease Burden According to 2021 Global Burden of Disease Data: Cross-Sectional Analysis

**DOI:** 10.2196/71468

**Published:** 2026-01-06

**Authors:** Aileen Park, Emily Woolhiser, Hannah Riva, Leo Wan, Haaris Kadri, Elizabeth Lamberty, Parker Juels, Sandra Jaroonwanichkul, Madison Reed, Catherine Hegedus, Dana Chen, Danielle Duffle, Jessica Kirk, Sydney Christensen, Emma Shelby, Robert Dellavalle

**Affiliations:** 1 School of Medicine, University of Colorado Anschutz Medical Campus, Aurora, CO, United States; 2 Kansas City University College of Osteopathic Medicine, Kansas City, MO, United States; 3 Memorial Hospital System, Pembroke Pines, FL, United States; 4 Paul L. Foster School of Medicine, Texas Tech University, El Paso, TX, United States; 5 West Virginia School of Osteopathic Medicine, Lewisburg, WV, United States; 6 School of Medicine, University of Missouri–Kansas City, Kansas City, MO, United States; 7 Kirksville College of Osteopathic Medicine, A.T. Still University, Kirksville, MO, United States; 8 Rocky Vista University College of Osteopathic Medicine, Parker, CO, United States; 9 A.T. Still University School of Osteopathic Medicine, Mesa, AZ, United States; 10 Department of Dermatology, School of Medicine, University of Minnesota, Phillips-Wangensteen Building, 516 Delaware St SE Suite 1-400, Minneapolis, MN, 55455, United States, 1-720-289-0247

**Keywords:** epidemiology, burden of disease, disability-adjusted life years, research funding, melanoma, psoriasis, dermatitis

## Abstract

**Background:**

Understanding the burden of various skin diseases can help guide funding allocation for skin disease research. A 2015 cross-sectional study found a partial correlation between US skin disease burden according to the 2010 Global Burden of Disease (GBD) study and National Institutes of Health (NIH) funding in 2012-2013.

**Objective:**

This study aims to identify trends, correlations, and disparities in US skin disease burden and NIH research funding allocation using the latest data from the GBD 2021 and NIH funding data from the fiscal years 2021-2022.

**Methods:**

A cross-sectional analysis was conducted to compare the disability-adjusted life years for 15 skin conditions from the GBD 2021 with NIH funding for these conditions in 2021-2022. Data were sourced from the GBD Results tool and the NIH RePORTER database.

**Results:**

NIH funding for skin disease research and US skin disease burden according to the GBD 2021 were partially correlated, with several outliers. Malignant skin melanoma and pruritus were relatively overfunded, while psoriasis and urticaria were relatively underfunded.

**Conclusions:**

Disease burden is just one of the many important factors that must be considered when allocating resources, including funding to encourage research efforts to improve patient outcomes and positively impact public health.

## Introduction

The Global Burden of Disease (GBD) study aims to quantify worldwide health losses due to a wide variety of illnesses and injuries [1]. Disease burden is one of many important factors guiding decisions on policy development, disease prevention initiatives, and research funding allocation [1,2]. The GBD study quantifies disease burden using disability-adjusted life years (DALYs), a measure that accounts for both mortality due to disease (years of life lost) and years lived with decreased health and quality of life (years lived with disability; YLDs) [1]. GBD also accounts for the severity of disability (defined by any short-term or long-term loss of health) attributed to the variety of illnesses and injuries included in the study by factoring disability weights into the calculation of YLDs [1,3].

Skin conditions are ubiquitous worldwide and affect millions each year. As a result, dermatology continues to be a consistently innovative field that makes large strides in patient care thanks to a heavy research focus. Public funding is a major contributor to research and innovation in this field. In 2015, Hagstrom and colleagues [4] conducted a cross-sectional study that found a partial correlation between US skin disease burden according to the GBD 2010 and National Institutes of Health (NIH) funding in the fiscal years 2012‐2013, identifying over- and underfunded diseases. Following this study, there have been major changes to the funding of dermatology research, with a 14.7% inflation-adjusted increase in research funding from 2015 to 2019 and fluctuations in funding after the COVID-19 pandemic [5,6]. This study reinvestigates the relationship between US skin disease burden using the latest GBD 2021 data and NIH funding data for 2021‐2022.

## Methods

### Overview

A cross-sectional analysis was conducted to compare DALYs for the 15 skin conditions included in the GBD 2021 with NIH funding for these conditions in 2021‐2022. Data were sourced from the GBD Results tool [1] and the NIH RePORTER database [7]. The search parameters used in GBD Results to obtain DALY metrics for all 15 aforementioned skin disease categories in the US were as follows: measure=“DALYs,” metric=“number,” location=“United States of America,” age=“all ages,” sex=“both,” and year=“2021.” DALY metrics were specifically gathered for the United States to facilitate a direct comparison between the US-specific burden of skin diseases measured by DALYs and funding allocated by the NIH in the United States for skin disease research.

To compile a comprehensive list of NIH-funded grants awarded for skin disease research during fiscal years 2021-2022, a total of 15 queries were entered into the NIH RePORTER database, with each query corresponding to one of the GBD skin disease categories. The following parameters were used to conduct all 15 of these search queries: fiscal year=“2021 and 2022,” text search logic=“advanced,” and limit project search=“project title, project terms, and project abstracts.” In the Text Search box, all *International Classification of Diseases, 10th Revision* codes categorized by the GBD 2021 under one specific skin disease category were strung with “AND,” “OR,” or “NOT” as determined necessary to capture all relevant NIH-funded grants.

All titles and abstracts of the grants obtained from NIH RePORTER were manually screened by two independent reviewers to determine inclusion versus exclusion (they were included if the grant studied any 1 of the 15 skin disease categories described by the GBD 2021). Following independent review, inclusion and exclusion decisions were cross-examined to identify conflicting decisions. A third reviewer served as a tie-breaker to resolve any discrepancies as needed.

Statistical analysis was performed assuming that the proportion of DALYs attributed to a disease should be the same as the proportion of NIH skin disease funding it receives (ie, if a specific disease is responsible for 25% of all US skin disease DALYs, that disease should receive 25% of all NIH skin disease funding). A one-to-one trendline was used to visualize this relationship and identify outliers representing relatively over- and underfunded skin diseases. An “observed-to-expected” ratio was calculated by dividing the true amount of funding a disease received by the amount of funding a disease could be expected to receive assuming a one-to-one relationship between DALYs and funding.

### Ethical Considerations

This study was exempt from review by the institutional review board, and no patient or participant consent was required or obtained, as this study did not constitute human subjects research and used publicly available data.

## Results

**Figure 1.**
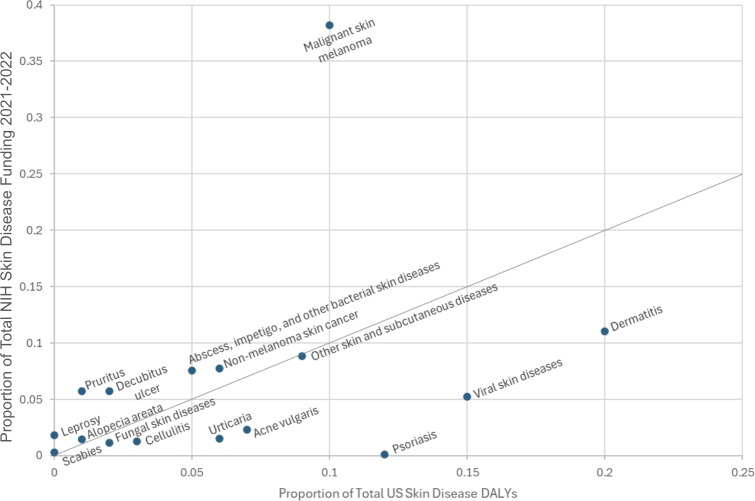
Scatterplot comparing proportion of National Institutes of Health (NIH) skin disease funding received in 2021‐2022 with the proportion of total US skin disease disability-adjusted life years (DALYs) according to the 2021 Global Burden of Disease study.

Our analysis revealed a positive correlation between the percentage of total US skin disease DALYs in 2021 and the percentage of total NIH skin disease funding in 2021‐2022. The correlation coefficient between these two data points was 0.3167 (95% CI 0.053626-0.579774). There were several key outliers when comparing DALYs to funding, indicating that certain skin diseases were relatively over- or underfunded in comparison to their proportion of total disease burden. Pruritus and malignant melanoma received 445% and 392% of the proportion of funding expected by their proportion of DALYs (Table 1). Other relatively overfunded diseases include leprosy, decubitus ulcers, bacterial skin diseases, and nonmelanoma skin cancer (Figure 1, Table 1).

**Table 1.** Comparison of disability-adjusted life year (DALY) rank from Global Burden of Disease GBD 2010 and 2021 study data, comparison of National Institutes of Health (NIH) funding in fiscal years 2012‐2013 (data from Hagstrom et al [4]) and 2021-2022 (data from the current analysis), and the percentage of total US skin DALYs (in 2021) and NIH skin disease funding (in 2021‐22).

Category	US DALY rank in 2021	Proportion of total US skin disease DALYs in 2021, %	US DALY rank in 2010^a^	NIH funding rank in 2021‐2022	Proportion of total NIH skin disease funding in 2021‐2022, %	NIH funding rank in 2012‐2013^a^	Observed-to-expected ratio for funding^b^
Pruritus	13	1.29	5	6	5.74	6	4.45
Malignant skin melanoma	4	9.75	3	1	38.19	1	3.92
Decubitus ulcer	11	1.87	8	7	5.74	11	3.07
Abscess, impetigo, and other bacterial skin diseases	8	4.90	13	5	7.56	9	1.54
Nonmelanoma skin cancer	6	6.10	2	4	7.73	2	1.27
Alopecia areata	12	1.36	11	12	1.48	13	1.09
Other skin and subcutaneous diseases	—^c^	9.30	—	3	8.87	3	0.95
Scabies	14	0.38	14	15	0.30	16	0.8
Dermatitis	1	19.98	1	2	11.03	5	0.55
Fungal skin diseases	10	2.17	9	14	1.14	10	0.53
Cellulitis	9	3.42	12	13	1.26	12	0.37
Viral skin diseases	2	14.61	6	8	5.22	4	0.36
Acne vulgaris	5	6.99	4	9	2.29	14	0.33
Urticaria	7	5.80	7	11	1.51	15	0.26
Psoriasis	3	12.10	10	16	0.09	7	0.0082
Leprosy^d^	15	0	15	10	1.84	8	—^d^

a Data obtained from Hagstrom et al [4].

b Percentage of funding vs percentage of DALYs.

c Not applicable.

d Ratio of funding proportion to DALY proportion could not be calculated for leprosy, as the proportion of DALYs for leprosy was 0.

Conversely, psoriasis, fungal skin diseases, cellulitis, urticaria, acne vulgaris, viral skin diseases, and dermatitis were underfunded. Notably, psoriasis received only 0.82% of the funding expected by its disease burden (Table 1). Funding for scabies, alopecia areata, and the “other skin/subcutaneous diseases” category appeared well matched to their disease burden, receiving between 80% to 110% of the funding predicted by their respective DALYs (Figure 1, Table 1).

## Discussion

### Principal Findings

This study reinvestigated the relationship between US skin disease burden and NIH skin disease research funding using the latest GBD 2021 data and NIH funding data from fiscal years 2021‐2022. Compared to Hagstrom et al’s [4] 2015 study, many of the same trends in relative over- and underfunding of skin diseases were observed. For example, malignant melanoma remains the most significantly overfunded skin disease relative to its disease burden (Table 1) [4]. Nonmelanoma skin cancer and leprosy also remain overfunded, while dermatitis, acne vulgaris, urticaria, fungal skin diseases, and cellulitis remain underfunded (Table 1) [4]. Interestingly, pruritus and decubitus ulcers, previously underfunded in 2015, now appear to be relatively overfunded (Table 1) [4]. Funding for psoriasis was well matched to its disease burden in 2015, but in our updated analysis, psoriasis is the most underfunded skin disease category. Similarly, viral skin diseases were well funded in 2015 and now appear underfunded (Table 1) [4].

It is important to consider disease burden when allocating research funding to ensure adequate resources are being directed toward diseases with the most significant impact. Dedicating more resources toward high-burden diseases can improve individual health and quality of life by driving the development of innovative treatments and can also provide long-term economic benefits by reducing health care costs and increasing overall workforce productivity.

In addition to disease burden, many other factors also significantly impact resource prioritization and funding allocation. For example, more research funding is likely to be allocated to diseases with strong public awareness and advocacy campaigns, such as malignant skin melanoma. Funding is also likely influenced by disease curability and the potential for therapeutic innovation. The NIH may also prioritize funding for diseases with lower incidence or prevalence but higher mortality (ie, metastatic melanoma, metastatic nonmelanoma skin cancer) rather than diseases with lower mortality but higher incidence or prevalence (ie, dermatitis and acne vulgaris).

### Limitations

It is important to keep in mind that using data strictly from the GBD study and the NIH does not fully capture all of the nuances of US skin disease burden and research funding. An important limitation of this analysis, similar to Hagstrom et al’s [4] prior study, is the exclusion of industry research funding by pharmaceutical companies and other nongovernmental entities from NIH funding data [4,7]. The NIH is the largest source of public funding for biomedical research; however, a significant portion of research funding also comes from nonprofits, philanthropic organizations, and private industry [8]. Therefore, while a disease may appear underfunded relative to its disease burden using GBD and NIH data alone, additional research funding from nongovernmental agencies may be filling this perceived gap in resource allocation. For instance, although our analysis showed that psoriasis received significantly less funding from the NIH relative to its disease burden, substantial funding from pharmaceutical companies has driven the development of innovative new drugs (ie, IL-23 and IL-17 inhibitors) that have transformed the treatment of psoriasis in recent years [9]. Similarly, previous reviews have cited US $22,291,506 in nonprofit funding for dermatology research in 2019 alone and US $9.3 billion dollars of private equity investment in dermatology health care and research between 2011 and 2021 [10,11].

### Conclusions

Given the wide variety of factors that must be considered in order to optimally allocate research funding, several guidelines may help ensure that funding is prioritized for research efforts that will guide clinical practice, improve patient outcomes, and positively impact public health. In addition to prioritizing high-burden diseases, prioritizing funding for translational research can help expedite the incorporation of knowledge gained from basic science research into clinical practice and patient care. Periodically evaluating the real-world impact of funded research using metrics including patient outcomes and cost-efficacy can also help ensure that funding is being distributed to research that is meaningfully impacting clinical practice. Increased funding for conditions that are impacting our patients will allow innovative solutions that improve patient quality of life. With these guidelines in mind, disease burden can easily be incorporated as one of the many important factors that should be used to inform research funding allocation, clinical practice guidelines, and health policy.

## Abbreviations

DALY: disability-adjusted life year
GBD: Global Burden of Disease
NIH: National Institutes of Health
YLD: year lived with disability
